# A Novel Method for Food Market Regulation by Emotional Tendencies Predictions from Food Reviews Based on Blockchain and SAEs

**DOI:** 10.3390/foods10061398

**Published:** 2021-06-17

**Authors:** Zhihao Hao, Guancheng Wang, Dianhui Mao, Bob Zhang, Haisheng Li, Min Zuo, Zhihua Zhao, Jerome Yen

**Affiliations:** 1 Beijing Key Laboratory of Big Data Technology for Food Safety, School of Computer, Beijing Technology and Business University, Beijing 100048, China; hao_zhihao@126.com (Z.H.); lihsh@th.btbu.edu.cn (H.L.); zuomin@th.btbu.edu.cn (M.Z.); 2 Department of Computer and Information Science, University of Macau, Macau 999078, China; wanggc@gdou.edu.cn (G.W.); jeromeyen@um.edu.mo (J.Y.); 3 National Engineering Laboratory for Agri-Product Quality Traceability, Beijing Technology and Business University, Beijing 100048, China; 4 College of Electronic and Information Engineering, Guangdong Ocean University, Zhanjiang 524088, China; 5 School of Law, China University of Political Science and Law, Beijing 102249, China; zhaozhihua@cupl.edu.cn

**Keywords:** blockchain, deep learning, food market, regulation

## Abstract

As a part of food safety research, researches on food transactions safety has attracted increasing attention recently. Food choice is an important factor affecting food transactions safety: It can reflect consumer preferences and provide a basis for market regulation. Therefore, this paper proposes a food market regulation method based on blockchain and a deep learning model: Stacked autoencoders (SAEs). Blockchain is used to ensure the fairness of transactions and achieve transparency within the transaction process, thereby reducing the complexity of the trading environment. In order to enhance the usability, relevant Web pages have been developed to make it more friendly and conduct a security analysis for using blockchain. Consumers’ reviews after the transactions are finished can be used to train SAEs in order to perform emotional tendencies predictions. Compared with different advanced models for predictions, the test results show that SAEs have a better performance. Furthermore, in order to provide a basis for the formulation of regulation strategies and its related policies, case studies of different traders and commodities have also been conducted, proving the effectiveness of the proposed method.

## 1. Introduction

Food plays a very important role in people’s daily life. Recently, the food industry has become one of the pillar industries of the national economy [1]. Food safety can be divided into food quality safety and food transaction safety. However, traditionally-related researches have focused on food quality and safety and have ignored the importance of food transaction safety. This situation has been improved in recent years: For example, food safety investment research has received increasing attention, as it contributes to the analysis of the distribution of decision rights in value chain organizations [2] and business relationship satisfaction for small- and medium-sized enterprises in agriculture food processing and exporting [3]. In addition, there is an increasing number of research on the food supply chain [4,5,6]. However, these researches are mostly focused on the food supply chain itself without considering the food transaction environment complexity, which is determined by the multiple stakeholders involved in the food transaction environment. Specifically, these stakeholders can be divided into farmers, processors, traders, wholesalers, retailers, and consumers. These roles can cause many problems such as information asymmetry, which may seriously affect the transaction process and lead to problems such as difficulty in tracing problematic products. In addition, the emotional changes of these roles are also one of the important variables affecting complexity. That being said, the current research does not pay enough attention to reviews data that can reflect food choices and preferences.

Blockchain provides a feasible solution for solving problems like information asymmetry due to its features, such as transparency, that cannot be tampered with and distributed storage. Recently, blockchain has been applied to the food industry by many large companies. For example, IBM and Wal-Mart use blockchain technology to establish a food traceability alliance. In addition, Wal-Mart is also cooperating with Nestlé to develop a global food traceability and management system based on blockchain. These projects have achieved the traceability of foods with problems to a certain extent, thus ensuring the credibility of traders and the fairness of transactions. However, the large amount of data stored in the blockchain has not been fully applied and mined. The lack of appropriate data analysis methods is one of the reasons for this issue.

The development of deep learning has brought opportunities for this. Deep learning is a sub-field of machine learning, which aims to imitate some work processed by the human brain, such as object detection [7] and speech recognition [8]. Simply speaking, deep learning models can learn by self-training and become one of the most representative methods to realize artificial intelligence (AI). Many companies have also begun to try to apply AI to the food field for improvements. For instance, McCormick, a seasoning company has decided to use AI to analyze the taste data collected over the past 40 years to predict the flavor of food so as to meet consumer preferences. Deep learning models can also be used for food recognition to help food manufacturers and farmers identify undernourished crops and detect diseases. In addition, deep learning can also provide strong technical support for the regulation of the food market: The results generated can provide a basis for the implementation of precise regulation.

Motivated by these, a consumer sentiment prediction method combining blockchain and deep learning models has been proposed to regulators for the formulation of regulatory measures in the food market. In order to establish a better explanation, the stakeholders involved in the food market can be divided into traders and consumers. Blockchain can provide a support platform for traders to complete transactions, where the related transactions information will be stored in the blockchain for all parties to access and view. The number of reviews with sentiment features are used as the data set for the deep learning model. Here, stacked autoencoders (SAEs) are used due to its excellent performance in prediction, which has been verified in the experiments. Overall, the highlights of this research can be divided into three parts.

- Blockchain has been used by different entities in the food transactions process, where it also can be used as a data hosting platform to ensure the authenticity of the data;
- The number of sentiment reviews are used as the data set for feeding the deep learning model. Here, the deep learning model can be used to predict the emotional tendencies of the consumers;
- Based on the predicted results, regulators can formulate corresponding measures to prevent things that are not conducive to market development.

The remaining structure is organized as follows. The theoretical background is discussed in Section 2. Section 3 presents the empirical framework. Method implementation is described in Section 4, while Section 5 shows the analysis of the experiments results and a case study. Finally, Section 6 concludes this paper.

## 2. Theoretical Background

Recently, blockchain technology has been greatly developed in the food field to prevent problems such as food fraud and achieve food traceability [9]. It is considered to be a promising technology to ensure food safety, such as achieving a transparent food supply chain [10] and improving performance of agri-food supply chains [11]. For food transactions safety, a blockchain-based food transaction system has been developed in [12] to ensure the transparency of the transactions, with the effectiveness of this system also verified in [13]. For food quality and safety, a blockchain-based visualization method according to [14,15] has been proposed to trace the source of problematic products and observe the flow of related risks [16]. The data in the blockchain can provide a basis for precise regulations, but there is currently a lack of suitable data analysis methods to maximize the effect of the data recorded in the blockchain.

Machine learning methods have received more attention in the food field. The development of deep learning models provides the possibility to solve these problems. In the natural language processing (NLP) field, many deep learning models such as recurrent neural network (RNN) [17], long short-term memory (LSTM) [18], and convolutional neural networks (CNN) [19] are used for the sentiment analysis of text. On this basis, improvements to existing technologies have also made the classification of sentimental text increasingly accurate. For example, Wang et al. [20] proposed a tree-structured regional CNN-LSTM model that can improve the performance of sentiment analysis based on the extraction and weighting of useful sentiment information in each region. The development of deep learning models in sentiment classification provides a basis for the prediction of emotional tendencies. However, most of the deep learning models are used for flow prediction currently, such as LSTM and gate recurrent unit (GRU) [21]. Due to the advantages in time series data processing, LSTM has been used to predict short-term traffic flow [22], which plays a very important role in traffic optimization control and navigation planning. In addition, Zhang et al. [23] used GRU combined with weather data to predict traffic flow. Moreover, [24] used the SAEs for traffic flow prediction because of features like the unsupervised learning and dimensionality reduction in data and evaluated the performance of the model at daytime and nighttime. They proved the prediction of traffic flow using a combination of multiple SAEs with different parameters suitable for different periods in real applications.

These applications of deep learning models in sentiment classification and flow prediction provides opportunities for the prediction of emotional tendencies: After obtaining the number of texts with sentiments, the tendencies can be obtained by comparing the number of positive and negative texts. Afterwards, regulators can formulate reasonable regulatory strategies and related policies based on the prediction results of emotional tendencies to prevent problems that are not conducive to market development.

## 3. Empirical Framework

**Figure 1:**
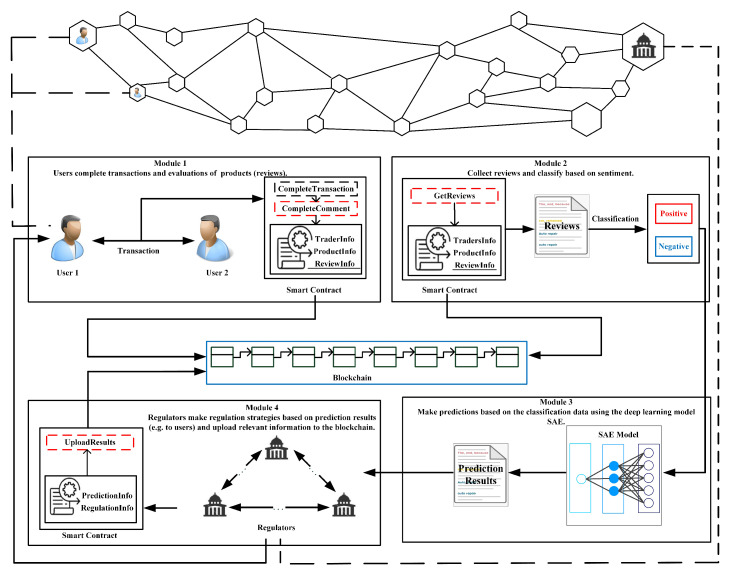
Framework of the proposed method.

Essentially, modern P2P technologies are fundamental to blockchain, which can achieve decentralized data sharing and storage. This enables any node in the blockchain network to view and access the data stored in the blockchain, where it can perform corresponding operations according to different permissions. Each transaction is completed after consensus is reached between nodes. The decentralized system runs on a virtual machine based on blockchain, which allows users to evaluate transactions and receive feedback through smart contracts independently. It can meet the various needs of users and integrate the work of regulatory agencies into the existing system at a lower cost, making it more effective. Based on these, the framework includes a few parts like Figure 1 shows.

As shown in Figure 1, the proposed method mainly involves two types of entities, which are users (traders) and regulators. Moreover, there are four modules included in the method: Transactions and reviews completion, reviews classification, sentiments predictions, and regulations based on the results of predictions. Applying these modules, the details of these two entities can be described as follows.

(1) Users can implement transactions in the blockchain network and then make reviews about the transactions. After the related reviews are finished, the relevant information can be stored in the blockchain after consensus. Due to the transparency of the blockchain, the data recorded in it can be used as training dataset to feed deep learning models for emotional tendencies predictions.

(2) Regulators can use SAEs to predict the emotional tendencies of users. Here, regulators can do regulations based on the results of predictions. For users who may have problems in the future, regulators can send warning messages to these users and make specific regulatory measures according to the outcome of these predictions.

All these entities exist in the blockchain network as nodes. The blockchain provides a more flexible framework, where the operations involved are transparent, which is conducive to efficient management and regulations in order to reduce transaction risks such as fraud. The important components about the blockchain in the proposed method consists of two parts:

(1) Blocks containing the data of transactions and reviews. Regulators can manage local records of relevant transactions, which are encrypted and assembled after a consensus. A cryptographic hash in each block points to the previous block, thereby the validation and traceability can be achieved. Blocks can be added into the blockchain chronologically. Due to this, all nodes have rights that access the data freely.

(2) Smart contracts are scripts stored on the blockchain, each of them has a unique address. A smart contract can be triggered by addressing a transaction. Then it executes automatically and independently on each node in the network in prescribed rules based on the data contained in the trigger transaction. After the transaction is completed, the smart contract and related transaction information and reviews are packaged into the block before being recorded in the blockchain.

Based on these, the detail process of the proposed method can be illustrated. Users participate in the transaction process as traders. After the transaction is completed, the trader can evaluate the transaction and make reviews by smart contracts, as shown in module 1 of Figure 1. Reviews can be divided into two parts: Consumers’ feelings about transactions (review content), and the evaluation of transactions (scores). Once the evaluation is completed, sentiment classification of the reviews is required, as shown in module 2. Since scores are an intuitive display of consumer emotions, reviews scores *c* correspond to different attitudes of the consumers (c∈[1,5], c∈Z). Here, we define the *c* in the range of [5, 3) as positive and in the range of (3, 1] as negative. Then the number of all positive and negative reviews can be collected respectively in a certain period of time, which can be used as the training dataset for a deep learning model (SAEs in module 3), where the SAEs predicts the corresponding number of reviews (positive or negative), so as to obtain the sentiment tendency. According to the results, regulators in module 4 can do regulations for users with negative sentiment tendency like send warning messages to remind users to make improvements. Through this method, it can strengthen regulation and maintain the sustainability of the market, allowing it to save considerably on labor and time costs.

## 4. Methodology of Emotional Tendencies Predictions

Emotional tendencies are handled by the deep learning model: SAEs. SAEs can be seen as stacks of autoencoders, which can be used as units to create a deep network [25].

### 4.1. Autoencoder

**Figure 2:**
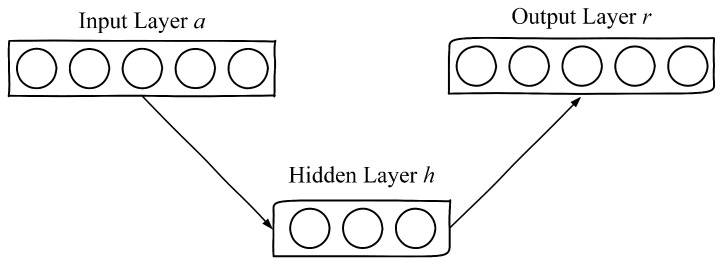
Schematic diagram of an autoencoder.

Autoencoders are a kind of neural network which attempts to replicate their inputs. As shown in Figure 2, they all have one input, hidden and output layers respectively. If there is a training dataset $A=a^{1},a^{2},a^{3},\ldots ,a^{n}$, where $a^{i}\in R^{d}$. For an autoencoder, it encodes an input $a^{i}$ to a hidden representation $h(a^{i})$ according to Equation (1), then it decodes $h(a^{i})$ and obtains a reconstruction representation $r(a^{i})$ by Equation (2).

(1) $$h\left(a\right)=f\left(M_{h}^{T}a+b\right)$$

(2) $$r\left(a\right)=g[M_{r}^{T}h\left(a\right)+b^{\prime }]$$

where $M_{h}^{T}$ and $M_{r}^{T}$ are the weight matrix between the input and hidden layers and decoding matrix between the hidden and output layers respectively, *b* and $b^{\prime }$ are bias vectors generated in encoding and decoding process. Here, f(x) and g(x) represent the logistic sigmoid function $\frac{1}{1+exp(-x)}$.

The error generated in reconstruction process also needs to be considered. Equation (3) is used for optimization by minimizing the reconstruction error L(A,R). The model parameters are defined as ψ and $\psi^{\prime }$:

(3) $$\psi ,\psi^{\prime }=\underset{\psi ,\psi^{\prime }}{\arg\min}L\left(A,R\right)=\underset{\psi ,\psi^{\prime }}{\arg\min}\frac{1}{2}\sum_{i=1}^{n}{∥a^{i}-r\left(a^{i}\right)∥}^{2}.$$

However, if the hidden layer size is equal to or larger than the input layer size, it may affect the training process of the autoencoders [26]. This problem can be solved by increasing the hidden units of nonlinear autoencoders or imposing restrictions like sparsity constraints [27]. After adding the sparsity constraints to the objective function, the autoencoder will consider the sparse representation of the hidden layer. In order to implement the sparse representation, a sparsity constraint $S_{con}$ can be achieved by minimizing the reconstruction error L(A,R) and the Kullback–Leibler (KL) divergence $D_{KL}\left(p|\right|q_{j})$ like Equation (4) shows:

(4) $$\begin{array}{cc} S_{con} & =L\left(A,R\right)+\zeta \sum_{j=1}^{N_{h}}D_{KL}\left(p|\right|q_{j})\\ \; & =\frac{1}{2}\sum_{i=1}^{n}{∥a^{i}-r\left(a^{i}\right)∥}^{2}+\zeta \sum_{j=1}^{N_{h}}\left[plog\frac{p}{q_{j}}+\left(1-p\right)log\frac{1-p}{1-q_{j}}\right]\\ \; & =\frac{1}{2}\sum_{i=1}^{n}{∥a^{i}-r\left(a^{i}\right)∥}^{2}+\zeta \sum_{j=1}^{N_{h}}\left(plog\frac{p}{q_{j}}+log\frac{1-p}{1-q_{j}}-plog\frac{1-p}{1-q_{j}}\right)\\ \; & =\frac{1}{2}\sum_{i=1}^{n}{∥a^{i}-r\left(a^{i}\right)∥}^{2}+\zeta \sum_{j=1}^{N_{h}}\left[p\left(log\frac{p}{q_{j}}-log\frac{1-p}{1-q_{j}}\right)+log\frac{1-p}{1-q_{j}}\right]\\ \; & =\frac{1}{2}\sum_{i=1}^{n}{∥a^{i}-r\left(a^{i}\right)∥}^{2}+\zeta \sum_{j=1}^{N_{h}}\left[plog\frac{p(1-q_{j})}{q_{j}\left(1-p\right)}+log\frac{1-p}{1-q_{j}}\right]. \end{array}$$

In Equation (4), the number of hidden units is represented by $N_{h}$, ζ is the sparity term weight, and *p* is a sparsity parameter that is close to zero. The average activation of hidden unit *j* over the training set can be defined as $q_{j}$, which is equal to $\frac{1}{N}\sum_{i=1}^{N}h_{j}\left(a^{i}\right)$. If $p=q_{j}$, $D_{KL}\left(p|\right|q_{j})$ is equal to 0, providing the sparsity constraint on the coding. This optimization problem can be solved by the backpropagation (BP) algorithm as well. The training process of an autoencoder is shown in Algorithm 1.
**Algorithm 1** Training process of an autoencoder.**Input:** Dataset $A=\left\{a^{i}\right\},a^{i}\in R^{KN}$, the number of hidden units $n_{h}$ and the number of the iterations *T*, initialize the matrices and biases randomly. **Output:** The optimization outcomes.
1:**for**i=1 to *T* **do**2:   Perform forward propagation to compute $r(a^{i})$.3:   Compute output error by $h\left(a^{i}\right)-r\left(a^{i}\right)$.4:   Perform backward propagation to compute Δψ and $\Delta \psi^{\prime }$.5:   Update ψ by ψ=ψ+Δψ and $\psi^{\prime }=\psi^{\prime }+\Delta \psi^{\prime }$.6:**end for**


As can be seen from Algorithm 1, $h(a^{i})$ of a trained autoencoder is regarded as the input vector’s feature vector, which will be used to extract the higher representation in SAE.

### 4.2. SAEs

**Figure 3:**
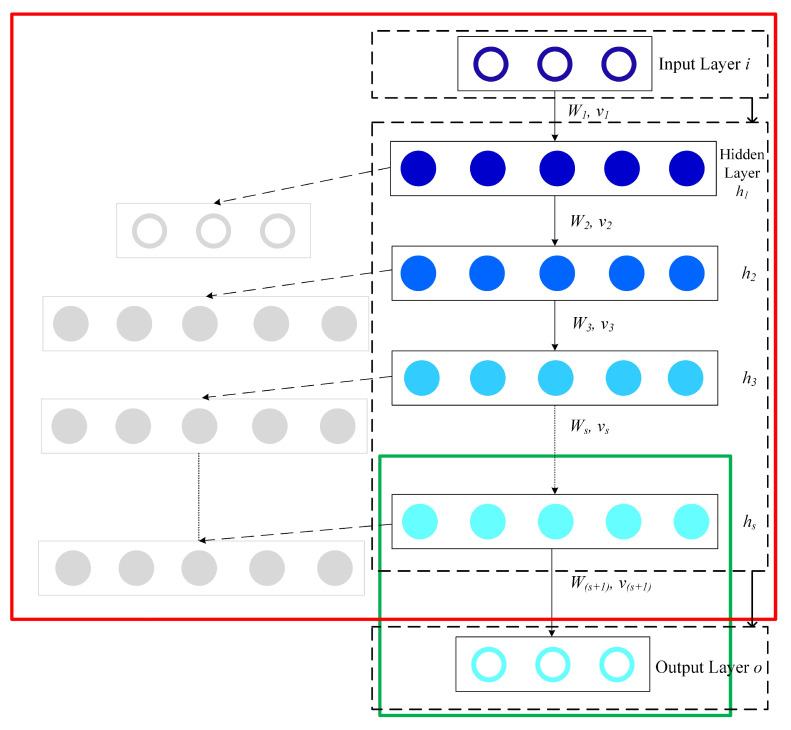
Layerwise training of SAEs.

**Figure 4:**
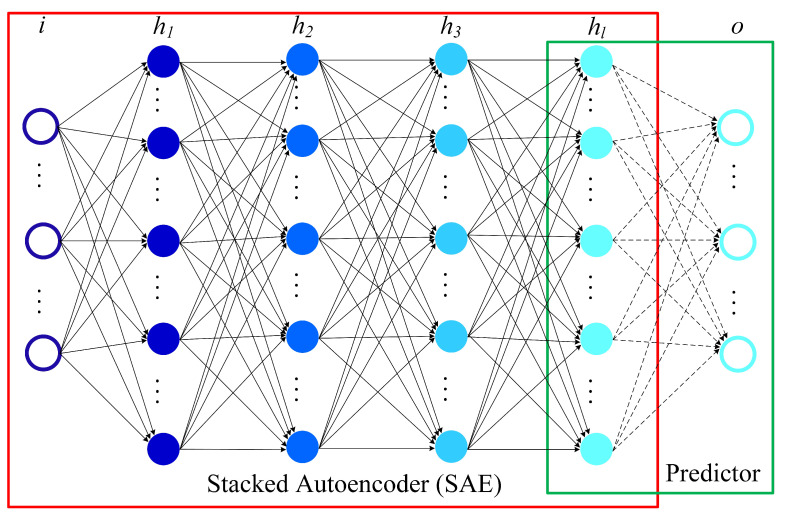
Deep architecture model for flow prediction. The SAE model is used for the extraction of flow features, with the logistic regression layer (predictor) used to do prediction.

A SAE model can be seen as stacks of autoencoders, where a deep network can be formed by the output generated using the previous layer as the current layer’s input [25]. As can be seen from Figure 3, a SAE model consists of a regression layer and multiple autoencoders. The SAE model is constructed by multiple autoencoders stacks hierarchically. For the purpose of flow prediction, the output layer (regression layer) can be used for fitting the output. The model architecture for flow prediction is comprised by SAEs and the predictor as shown in Figure 4. The model feed forward to do calculation for the output prediction corresponding to an input vector *a* by Equation (5) is:

(5) $$h_{s}=\left\{\begin{array}{cc} f(M_{1}^{T}a+b_{1}), & s=1\\ f(M_{s}^{T}h_{s-1}+b_{s}), & N_{h}\geq s=1 \end{array}\right.$$

(6) $$z_{s}=f\left(M_{N_{h}+1}^{T}h_{N_{h}+1}+b_{N_{h}+1}\right)$$

where $h_{s}$ represents the *s*^th^ hidden representation. $M_{s}$ is the weight matrix between the *s*^th^ hidden layer and the previous layer, $M_{s+1}$ is the weight matrix between the output layer and the $N_{h}$^th^ hidden layer. The bias vector of the *s*^th^ hidden layer and the output layer can be defined as $b_{s}$ and $b_{N_{h}+1}$ respectively.

### 4.3. Training Process of SAEs

The training of SAEs can be carried out through the combination of a backpropagation method and a gradient-based optimization technique. However, this training method will cause problems such as poor model performance. In order to solve this problem, a greedy layerwise unsupervised learning algorithm has been developed in [28] that can train neural network without these issues. The core of this method is to pretrain layers of a neural network in a hierarchical way. After the process of pretraining, backpropagation can be used to tune the parameters of the models to achieve better prediction results. The whole training process is shown in Algorithm 2.

### 4.4. Sentiment Tendency Determination

The sentiment tendency of a particular product of a trader at a certain moment can be obtained by comparing the predicted results of positive and negative reviews. Specifically, if the number of positive reviews at a certain moment is greater than the number of negative reviews, the sentiment tendency at this moment can be determined to be positive. On the contrary, the sentiment tendency can be determined as negative. Regulators can regulate specific traders based on the determination results of the emotional tendencies and traders can also make improvements to specific commodities based on the determination results, as shown in Algorithm 3.
**Algorithm 2** Training process of SAEs.**Input:** Training Dataset $A=\left\{a^{i}\right\},a^{i}\in R^{KN}$ the expected number of hidden layers $N_{h}$, the number of the pretraining iterations $T_{1}$. **Output:** The optimization results. 1:Set the ζ, *p* and initialize *M* and *b* randomly.2:Greedy layerwise training hidden layers.3:Training the first layer using Algorithm 1, where the input dataset is $A=\{a^{i}\}$.4:**for**s=2 to $N_{h}$ **do**5:   The output the hidden layer can be used as the input of the next layer.6:   Encoding parameters ${\left\{M_{1}^{s+1},b_{1}^{s+1}\right\}}_{s=0}^{N_{h}-1}$ can be found for (s+1)th hidden layer by minimizing the objective function.7:**end for**8:$\left\{M_{1}^{N_{h}+1},b_{1}^{N_{h}+1}\right\}$ can be initialized randomly.9:**for**i=1 to $T_{1}$ **do**10:   Use the backpropagation method with the gradient-based optimization technique to adjust parameters of the whole neural network hierarchically (from top to bottom).11:**end for**


**Algorithm 3** Process of sentiment tendency determination and regulations.**Input:** Number of positive reviews $n_{pos}$ and negative reviews $n_{neg}$ collected in the previous period, total period $T_{2}$, time interval *t*, identification numbers of traders $n_{ID}$, and total number of traders $N_{t}$. **Output:** Prediction results $P_{pos}$, $P_{neu}$, and $P_{neg}$
1:Collect reviews from traders.2:Do sentiment analysis and classification then get $n_{pos}^{\prime }$ and $n_{neg}^{\prime }$ generated in *t*.3:Feed ($n_{pos}$+$n_{pos}^{\prime }$) and ($n_{neg}$+$n_{neg}^{\prime }$) to SAEs and training through Algorithm 2 then obtain prediction results $N_{pos}$ and $N_{neg}$.4:**for**$n_{ID}$ to $N_{t}$ **do**5:   **for** i=0; $i<T_{2}$; i=(i+t) **do**6:    **if** $N_{pos}>N_{neg}$ **then**7:     Sentiment tendency is positive, result is $P_{pos}$.8:    **else**9:     **if** $N_{pos}=N_{neg}$ **then**10:      Sentiment tendency is neutral, result is $P_{neu}$.11:     **else**12:      Sentiment tendency is positive, result is $P_{neg}$.13:      Do regulations to trader $n_{ID}$.14:     **end if**15:    **end if**16:   **end for**17:**end for**


## 5. Experiments and Performance Analysis

### 5.1. Data Source

Reviews stored in the platform can be crawled through the website crawlers and smart contracts. However, due to limitations such as the throughput of the blockchain network, the amount of reviews collected from the test platform is small (less than 2000) and cannot be used as a training data set for the model. Therefore, in order to increase the amount of training data to ensure the quality of the generated results, Amazon Food Review [29] has been used as the main training data set. The time span of data collection is more than 10 years (October 1999–October 2012). A total of 568,454 reviews with about 74,258 products are included, which was developed by 256,059 users. After data processing like data cleaning, the reviews can be classified based on scores of products and reviews can be obtained with two sentiments (positive and negative). Next, we sort out the relevant reviews data according to the timestamp and the different number of sentiments reviews are counted in 5-min intervals of each other for each traders’ product. The number of positive and negative reviews collected in each of the three months can be used for the relevant predicted number of reviews on the first day after these three months: The data of the first two months can be chosen as the training dataset and the remaining one month’s data can be selected as the testing dataset.

Before feeding the dataset to the SAEs, the input layer size, the hidden number of layers, and the hidden number of units for each hidden layer needs to be determined. Due to the temporal correlations of the dataset, the proposed method considers the temporal and spatial relationship of the number of reviews: In order to predict its number at interval time $t_{j}$, the collected data at the previous time intervals ($A^{t_{j}-1},A^{t_{j}-2},\ldots ,A^{t_{j}-k}$, k∈[1,12]) can be used. The input space dimension can also be defined as $d_{i}$ and the output dimension is $d_{o}$, where $d_{o}$ is equal to the number of traders used in the experiments.

In order to provide a more accurate basis for better regulation, the proposed model has been set to the number of predicted reviews generated in 60 minutes, with the time ranging from 0:00 to 23:59. Based on the best architecture test for different prediction tasks in [26], the architecture of the proposed model consists of four hidden layers and the hidden units number is [300, 300, 300, 300], respectively in each hidden layer.

### 5.2. Metrics and Performance Analysis

In order to evaluate the performance of the proposed model, two metrics can be used here, which are MAE (mean absolute error) and RMSE (root mean squared error), correspondingly. These two metrics can be defined as below:

(7) $$MAE=\frac{1}{m}\sum_{i=1}^{m}\left|n_{i}-\hat{n_{i}}\right|$$

(8) $$RMSE=\sqrt{\frac{1}{m}\sum_{i=1}^{m}\left(\right|n_{i}-\hat{n_{i}}{\left|\right)}^{2}}$$

where $n_{i}$ is the collected number of reviews and $\hat{n_{i}}$ is the predicted number of reviews.

**Figure 5:**
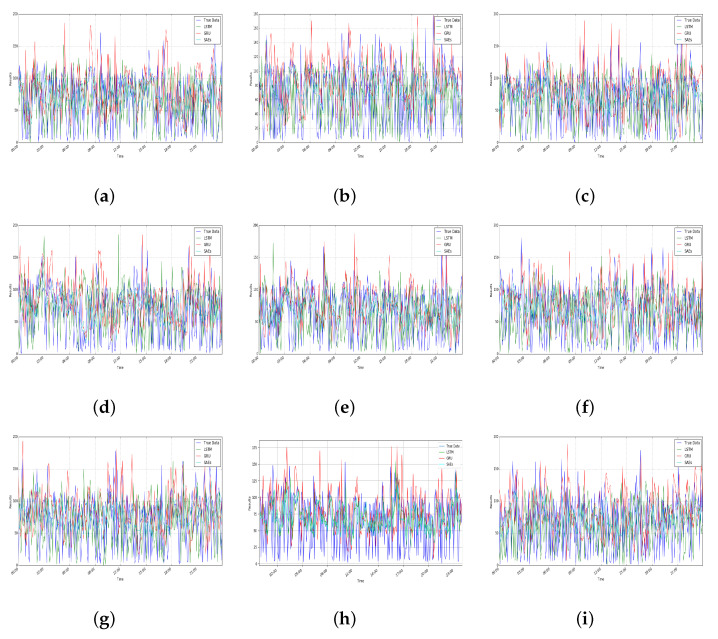
Prediction results on positive reviews.

**Figure 6:**
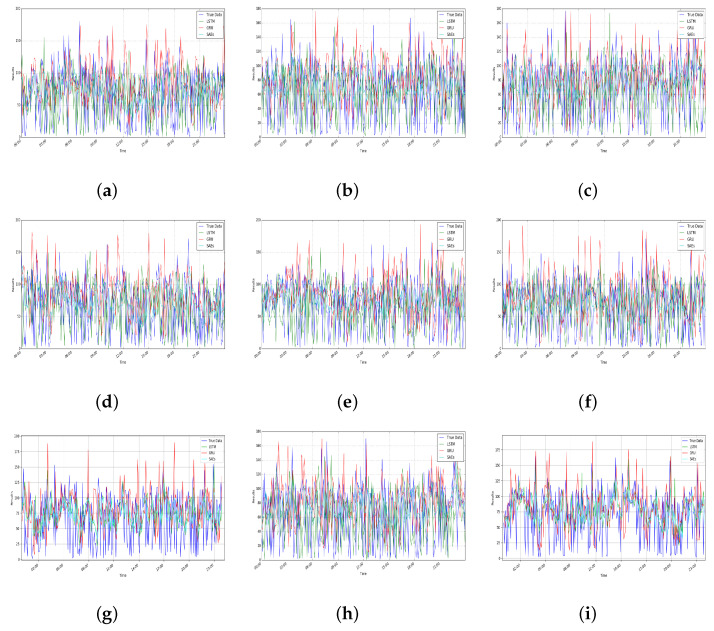
Prediction results on negative reviews.

For a certain product of a trader, Figure 5 and Figure 6 show the output of the deep learning model for the number of predicted reviews with a sentiment of positive and negative. In order to test its performance, the SAEs model has been compared with different deep learning models, LSTM and GRU, which are both advanced models for prediction [21]. In addition, the actual number of reviews (True Data in the figures) is also included. In all cases, the same data set has been used for performance comparison in the different models. The number of reviews data has only been used as the input for prediction without considering factors caused by something unrelated to commodities, such as the transportation process. It can be seen from Figure 5 and Figure 6, the number of predicted reviews has overall similar patterns with the true data. However, different models have different deviations. It can be seen from Table 1 that SAEs have low MAE values, which means the prediction accuracy is acceptable and comparable with the reported results (according to the previous work in [26], the mean relative error of the SAEs is less than 6.21%).

In addition, as shown in Table 1, LSTM and GRU both show similar performances on the number of predicted positive and negative reviews. Compared with these two models, SAEs have better performance. For positive reviews, SAEs increased by 5.114802 and 6.936406, respectively on the average values of MAE and RMSE compared to LSTM. Compared with GRU, SAEs increased 5.632776 and 8.206668 on MAE and RMSE, correspondingly. As for negative reviews, compared with LSTM and GRU, SAEs improved on MAE and RMSE by 5.138940, 5.551480, 6.850650, and 8.171320, respectively. This also demonstrates that SAEs are more accurate than LSTM and GRU for 60 minutes predictions and because of the lower value of MAE and RMSE, the results generated by SAEs are more closer to the true data. Overall, the SAEs model for the number of predicted reviews is effective.

### 5.3. Case Study

**Figure 7:**
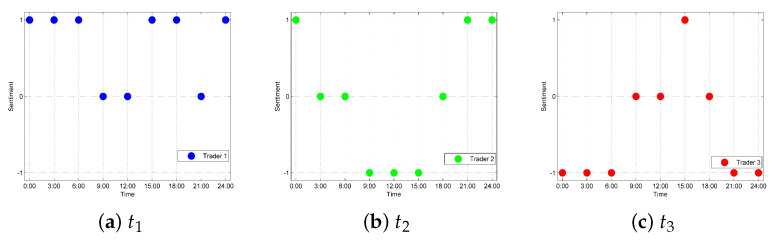
Sentiment tendency for different traders.

**Figure 8:**
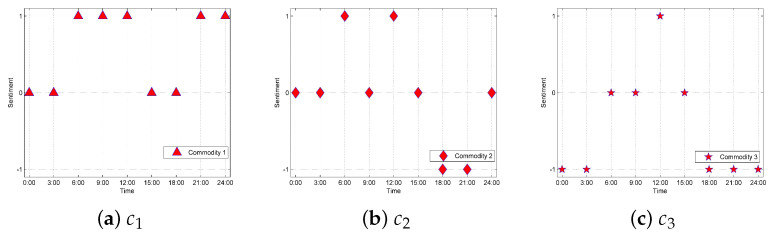
Sentiment tendency for $t_{3}$’s different commodities.

For a specific trader or commodity, the sentiment tendency at a certain moment can be determined by the prediction results comparison. For further analysis, we selected different traders $t_{1}$, $t_{2}$, and $t_{3}$ for a case study. In addition, for the different commodities $c_{1}$, $c_{2}$, and $c_{3}$ of $t_{1}$, we analyzed the relevant emotional tendencies to help $t_{1}$ make improvements. In order to better analyze the results, we set positive, neutral, and negative sentiment tendencies as 1, 0, and −1, respectively as shown in Figure 7 and Figure 8.

#### 5.3.1. For Regulations of Traders

For regulators, the sentiment tendency can provide a basis for market regulations. Specifically, regulators will send warning messages to the traders once the emotional tendencies can be judged as negative at a certain moment. As shown in Figure 7b, $t_{2}$ will receive warning messages at 9:00, 12:00, and 15:00. In addition, traders with more negative emotional tendencies will be listed as key targets for regulation. Regulators can take more specific measures for these key targets, such as suspending business for rectification. As shown in Figure 7c, due to more negative emotional tendencies for $t_{3}$, $t_{3}$ will receive warning messages at 0:00, 3:00, 6:00, 21:00, and 23:59, while $t_{3}$ will also receive a message that lists them a key regulation target at 23:59. Only if $t_{3}$ makes corresponding improvements to increase consumer satisfaction and reduces the occurring number of negative tendencies, the relevant measures will be cancelled. This approach can increase consumer satisfaction and avoid trader speculation, thereby maintaining the sustainable development of the market.

#### 5.3.2. For Improvements of Commodities

For traders, consumer emotional tendencies about commodities are the basis of relevant improvements. The emotional tendencies is essentially the accumulation of consumers’ emotional tendencies towards related commodities. Therefore, the research on the emotional tendencies of commodities is very important for traders. Figure 8 lists three commodities $c_{1}$, $c_{2}$, and $c_{3}$ of $t_{3}$ for further analysis. It can be seen from Figure 8 that $c_{1}$ does not have negative tendencies and there are more positive tendencies, such that it can be inferred that $c_{1}$ has better sales and higher consumer satisfaction. The number of positive and negative emotional tendencies of $c_{2}$ are equal, with most of the time periods neutral, indicating that the sales and consumer satisfaction of $c_{2}$ are average. For $c_{3}$, the number of positive tendencies is small and the negative tendencies occupy a majority, which implies that most consumers are not satisfied with $c_{3}$. Therefore, it is recommended that $t_{3}$ expand the purchase and sales scale of $c_{1}$ and reduce the scale of $c_{2}$ and $c_{3}$ accordingly. In addition, the related negative reviews content of $c_{3}$ can be used for analysis and to make improvements for dissatisfied consumers like product quality. In this way, traders can clearly know the problems of their commodities with low consumer satisfaction, so that they can make improvements in future sales. Moreover, through the analysis of emotional tendencies, traders will also know which commodities are more popular, allowing them to expand the scale of these products in the future to increase profits and further improve consumer satisfaction, while increasing the number of positive tendencies.

## 6. Conclusions

### 6.1. Discussion and Theoretical Advance

Sentiment tendencies of the consumers can reflect their preferences. It can help to understand their food choices, which can satisfy consumers needs and provide food companies with a competitive advantage. More and more researchers have realized this [30,31,32,33]. For example, Zhang et al. [34] conducted an online survey for dwellers in 489 cities in Australia. The results showed that consumers value various food attributes arranged in a hierarchical order, and there is significant heterogeneity in consumers’ food preferences. In addition, the significance of this research for food regulatory agencies and companies was discussed: risk management through food preference research is essential for building consumer confidence in food safety. However, these researches are mostly surveys of consumers in a specific cultural and economic development environment. Therefore, there are limitations: these surveys cannot meet the complexity of the actual food trading environment. In addition, the methods used in these studies are also important factors that affect the results of their analysis. All of these have brought challenges to the effective implementation of market regulation.

Recently, deep learning has been widely used in market regulation due to its good performance in data processing. For example, Jahangir et al. [35] proposed a new clustering method based on deep learning to manage the energy market through behavior modeling of electric vehicles. Fu et al. [36] used a deep learning method to predict online lending default for financial market regulation, with the results showing that the method is effective. However, there are very few studies on applying deep learning to food market regulation. Most of the research is focused on the food analysis. For example, Fuentes et al. [37] modeled Pinot Noir aroma profiles by machine learning algorithms based on management information data of water and weather. Gunaratne et al. [38] used machine learning methods and Near Infra-red to evaluate chocolate quality based on chemical fingerprints. Most of these researches focus on the analysis of the physical and chemical indicators [39,40], lacking any direct connection to consumer behavior such as sentiment analysis. In addition, there are some problems like high training costs and poor adaptability, which have brought uncertainty to these methods.

In order to meet all these challenges, this research uses current advanced technologies to provide a solid foundation for the healthy development of the food market: it can objectively reflect the food preferences of consumers in the market and provide a basis for the formulation of regulatory strategies. The deep learning model has been used to analyze the emotional tendencies of consumers, and the analysis results can be used as a starting point for regulation strategies and its related policies formulation. These policies are not only conducive to regulating the order of the food market, but also can prevent traders from using improper methods to seek economic benefits. In addition, due to the development of blockchain in the food industry [12,13,41], it can be used to eliminate defects in the food market such as information asymmetry and effective consumer needs that can-not be satisfied, thereby ensuring the effectiveness of the policies in many aspects like eliminating protectionism and promoting the unification of market rules. Moreover, the policies formulated can fully stimulate the potential of the market and promote beneficial competition among traders, thereby realizing self-adjustment and sustainable development of the food market [42].

Specifically, in order to prevent possible risks occurring in the food market and to ensure the safety of food transactions, this paper proposes a method that combines blockchain and a deep learning model to predict and determine the emotional tendencies of consumers, thus providing a basis for the formulation of regulatory strategies and its related polices. The advantage of the proposed method is that it not only guarantee the validity of the data but also analyze the data well: the blockchain is used to ensure the authenticity of the reviews, while SAEs are used to predict consumer sentiment based on these reviews. Performance tests show that the SAEs perform better than LSTM and GRU. Furthermore, case studies can prove that the predicted emotional tendencies are conducive to formulating reasonable regulatory strategies to avoid related risks. Therefore, the proposed method can meet the current challenges well both in terms of technology and performance. In summary, the proposed method can help traders improve the food transaction process, thereby creating a fair and orderly trading environment.

### 6.2. Limitations and Further Researches

Limited by the throughput and block generation speed of the blockchain, the current research has some limitations in data storage time. To resolve this issue, we will try to use a higher configuration network to increase the storage speed in future. In addition, the improvement of the SAEs is also one area we wish to work on in the future; although the SAEs obtained a better performance, the predicted results still have slight deviations. In future, we will improve the prediction accuracy by improving the model structure and increased processing of the data set. Moreover, the proposed method still has some limitations in practicability: The size of the data set affects the training results of the proposed method. At present, large-scale data can only be easily obtained by regulatory authorities and large companies. For most of small- and medium-sized enterprises in the market, which cannot obtain such a large data set, in future we will try to use training models suitable for small data sets such as reinforcement learning to improve the training effects. For consumer behaviors that may affect the market, we will further explore the development of consumer information frameworks and presentations based on consumer food selection criteria and emotional changes caused by different foods. Improvements and development in all of these areas will be the focus of our future research work. 

## Figures and Tables

**Table 1:** Metric results on prediction, where (a)–(i) correspond to Figure 5 and Figure 6.

		LSTM		GRU		SAEs	
		MAE	RMSE	MAE	RMSE	MAE	RMSE
Positive	(a)	36.335451	46.211931	43.180532	53.695487	36.611738	44.703110
	(b)	44.001377	54.172575	42.477046	52.547250	37.151156	45.119240
	(c)	43.429057	53.584650	42.236403	52.846026	37.062104	44.879368
	(d)	43.931223	53.855806	43.567233	54.203652	37.256250	45.128175
	(e)	43.798400	53.697382	42.014015	52.638279	36.890818	44.813605
	(f)	42.934769	52.923344	43.011107	53.428688	37.135335	45.199576
	(g)	43.019526	53.086045	42.399551	53.085417	36.812493	44.660683
	(h)	37.742285	46.050368	42.803237	53.571811	36.928434	45.036694
	(i)	43.912802	53.713543	42.077536	52.711389	37.223346	45.327537
	AVE	42.122766	51.921738	42.640740	53.192000	37.007964	44.985332
Negative	(a)	43.184724	53.525993	42.811964	53.476943	36.864293	44.842421
	(b)	44.018736	53.891109	42.662726	53.234308	37.159017	45.017255
	(c)	43.014621	52.914675	42.347422	52.891398	37.109032	44.888128
	(d)	43.707601	53.563837	42.786897	53.127477	37.048903	44.897605
	(e)	44.223738	54.368301	42.847674	53.379149	37.307459	45.169035
	(f)	42.831805	53.116795	42.697464	53.403691	37.135870	45.198482
	(g)	37.823529	46.089258	42.632475	53.294239	37.181486	45.212046
	(h)	43.407516	53.513330	42.295810	52.858094	36.573601	44.688961
	(i)	37.737973	45.994387	42.580679	53.198429	37.320087	45.407901
	AVE	42.216690	51.886410	42.629230	53.207080	37.077750	45.035760
